# Factors affecting mortality following emergency admission for chronic obstructive pulmonary disease

**DOI:** 10.1186/1471-2466-14-151

**Published:** 2014-09-24

**Authors:** Wakae Hasegawa, Yasuhiro Yamauchi, Hideo Yasunaga, Mitsuhiro Sunohara, Taisuke Jo, Hiroki Matsui, Kiyohide Fushimi, Kazutaka Takami, Takahide Nagase

**Affiliations:** Department of Respiratory Medicine, Graduate School of Medicine, The University of Tokyo, 7-3-1, Hongo, Bunkyo-ku, 113-8655 Tokyo, Japan; Department of Clinical Epidemiology and Health Economics, School of Public Health, The University of Tokyo, 7-3-1, Hongo, Bunkyo-ku, 113-8655 Tokyo, Japan; Division for Health Service Promotion, The University of Tokyo, 7-3-1, Hongo, Bunkyo-ku, 113-8655 Tokyo, Japan; Department of Health Policy and Informatics, Tokyo Medical and Dental University Graduate School of Medicine, 1-5-45, Yushima, Bunkyo-ku, 113-0034 Tokyo, Japan

**Keywords:** Chronic obstructive pulmonary disease, All-cause in-hospital mortality, Activities of daily life, Emergency hospital admission

## Abstract

**Background:**

Chronic obstructive pulmonary disease (COPD) is a major cause of mortality worldwide. Patients with COPD frequently have systemic comorbidities that often require unscheduled hospitalization for exacerbation and deterioration of physical conditions, and can have a poor prognosis. We verified factors affecting patients’ short-term mortality, using a national inpatient database in Japan.

**Methods:**

We retrospectively collected data for COPD patients (age: >40 years) with emergency admission between July 2010 and March 2013, using the Diagnosis Procedure Combination database. We performed multivariate logistic analyses fitted with a generalized estimating equation to assess factors associated with all-cause in-hospital mortality.

**Results:**

A total of 177,207 patients (mean age: 77.5 years; males: 72.9%) were identified. All-cause in-hospital death occurred in 23,614 patients (13.7%). Higher mortality was associated with older age, male sex, lower body mass index, more severe dyspnea, lower level of consciousness, and worse activities of daily life. Higher mortality was also associated with comorbid conditions, including bacterial pneumonia, aspiration pneumonia, interstitial pneumonitis, pulmonary embolism, respiratory failure, lung cancer, heart failure, cerebral infarction, liver cirrhosis, and chronic renal failure.

**Conclusions:**

Our study demonstrated that all-cause in-hospital mortality in patients with COPD who required emergency hospitalization was associated with deteriorated general conditions and comorbidities at admission. Physicians should take into account these prognostic factors to choose better treatment options for COPD patients.

## Background

Chronic obstructive pulmonary disease (COPD) is a major cause of chronic morbidity and mortality throughout the world, and the fourth cause of death worldwide [1, 2]. COPD is characterized by persistent airflow limitation and is usually progressive [3]. The characteristic symptoms of COPD are cough, productive sputum, and dyspnea on exertion, which lead to poor activities of daily life (ADL) and decreased health-related quality of life. Patients with COPD often require emergency hospital admission because of acute exacerbations of COPD, respiratory failure, and deterioration of general conditions, which would threaten their lives [4].

Moreover, COPD often coexists with other systemic comorbid diseases, such as lung cancer, cardiovascular disease, osteoporosis, and diabetes mellitus [5]. These comorbidities have shared risk factors with COPD, and can be considered to influence one another. The comorbidities in COPD would also become exacerbated, require emergency hospitalization of the patients, and affect their mortality [6].

Evaluation of mortality after emergency admission of patients with COPD, including acute exacerbations of COPD and their comorbid diseases, is important and could lead to a better prognosis for the patients through the induction of appropriate interventions. However, there are few available data regarding all-cause mortality in these patients, although there are several studies on the mortality of patients with acute exacerbations of COPD [7, 8].

In this study, we aimed to evaluate the short-term all-cause in-hospital mortality of patients with COPD who required emergency hospital admission not only for exacerbations of COPD, but also for any cause. We tried to verify factors, including patients’ general conditions and comorbidities at admission, that affect the mortality after emergency admission. The obtained results suggest the importance of understanding the patients’ general conditions and comorbidities at admission.

## Methods

### Diagnosis Procedure Combination (DPC) database

The DPC database is a nationwide administrative inpatient database in Japan, and includes administrative claims data and discharge abstract data. The main diagnosis, comorbidities present at admission, and complications occurring during hospitalization are coded using International Classification of Disease and Related Health Problems 10th Revision (ICD-10) codes accompanied by text data in Japanese. The database also contains the following patient details: age; sex; body height and weight; severity of dyspnea based on the Hugh-Jones Dyspnea Grade [9] at admission; levels of consciousness based on the Japan Coma Scale [10, 11] at admission and discharge; status of disability of ADL based on the Barthel Index [12, 13] at admission; and discharge status, including in-hospital death.

Grading of dyspnea severity based on the Hugh-Jones classification [9] was defined as follows: I, patient’s breathing is as good as that of other people of their own age and build during work, walking, and climbing hills or stairs; II, patient is able to walk at pace with normal people of their own age and build on level ground, but is unable to keep up on hills or stairs; III, patient is unable to keep up with normal people on level ground, but is able to walk about a mile or more at their own speed; IV, patient is unable to walk more than about 50 yards on level ground without a rest; V, patient is breathless on talking or undressing, or unable to leave their house because of breathlessness; Unspecified, patient is unable to be classified into the above grades because of bedridden status. The level of consciousness on the Japan Coma Scale [10, 11] was defined as follows: one-digit codes (1–3) are attributed to patients who are awake without any stimuli; two-digit codes (10–30) are attributed to patients who can be aroused by some stimuli; three-digit codes (100–300) are attributed to those with coma. The Japan Coma Scale and Glasgow Coma Scale assessments are well correlated [14].

This study was approved by the Institutional Review Board of The University of Tokyo, which waived the requirement for patient informed consent because of the anonymous nature of the data.

### Patient selection

We retrospectively collected data for patients aged over 40 years who had been admitted to hospital because of COPD (ICD-10 codes: J41, J42, J43, J44) as the main disease, or who had been admitted for any cause, but had COPD as a comorbidity, required emergency hospital admission, and were discharged between 1 July 2010 and 31 March 2013.

### Comorbidities

The following comorbidities were identified using ICD-10 codes: pneumonia caused by pathogenic microbes (J10–J18); aspiration pneumonia (J690); interstitial pneumonia (J84); pulmonary embolism (I26); respiratory failure (J96); lung cancer (C34); heart failure (I50); bone fracture (S02, S12, S22, S32, S42, S52, S62, S72, S82, S92, T02, T10, T12); cerebrovascular diseases (I60–I69); liver cirrhosis (K703, K74); chronic renal failure (N18).

### Outcome

The outcome of this study was all-cause in-hospital mortality in patients with COPD who required emergency hospital admission.

### Statistical analysis

The chi-square test was used to compare in-hospital mortality between the groups. A multivariate logistic regression for in-hospital mortality was performed to analyze patient-level factors associated with the outcome after adjustment for within-hospital clustering using a generalized estimating equation [15]. The threshold for significance was a value of P < 0.05. All statistical analyses were performed using SPSS version XX (IBM, Somers, NY).

## Results

### Patient characteristics

Among 19 million patients recorded during the 33 months between July 2010 and March 2013, we identified 172,707 patients with COPD who were aged over 40 years and required emergency hospital admission to DPC-participating hospitals. Among them, the number of patients admitted primarily because of COPD was 18,487, and the number of patients admitted because of other causes, but with COPD as a comorbidity, was 154,220. The mean age was 77.5 (standard deviation (SD) 9.8) years in total, 77.2 (SD 9.4) years for males, and 78.5 (SD 11.1) years for females. The mean body mass index (BMI) was 20.1 (SD 3.9) in total, 20.1 (SD 3.8) for males, and 20.1 (SD 4.6) for females. The median length of hospital stay was 16 (interquartile range (IQR) 10–29) days.

### All-cause in-hospital mortality in patients with COPD who required emergency hospital admission

Overall, 23,614 patients (13.7%) died in hospital after emergency hospital admission. The median time from admission to death was 19 (IQR 6–42) days. Table 1 shows the relationships between in-hospital mortality and patients’ general conditions and comorbidities. All-cause in-hospital mortality in patients with COPD was associated with older age, male sex, lower BMI, more severe dyspnea by the Hugh-Jones Dyspnea Grade, lower level of consciousness by the Japan Coma Scale, and worse disability of ADL by the Barthel Index. In addition, higher mortality in patients with COPD was associated with comorbid diseases, including bacterial or viral pneumonia, aspiration pneumonia, interstitial pneumonitis, pulmonary embolism, respiratory failure, lung cancer, heart failure, cerebrovascular diseases, liver cirrhosis, and chronic renal failure.

**Table 1:** **All-cause in-hospital mortality in patients with COPD who required emergency hospitalization**

	Total (n)	Death (n)	Mortality (%)	p-value
Age (yr)				<0.001
40–49	2,055	71	3.5	
50–59	6,241	418	6.7	
60–69	25,359	2,241	8.8	
70–79	57,127	7,137	12.5	
80–89	68,092	11,144	16.4	
90–99	13,511	2,541	18.8	
>100	322	62	19.3	
Sex				<0.001
Male	136,632	19,858	14.5	
Female	36,075	3,756	10.4	
BMI				<0.001
<18.5	55,376	9,399	17	
18.5–22.9	61,183	6,310	10.3	
23.0–24.9	16,190	1,275	7.9	
25.0–29.9	12,970	920	7.1	
>30.0	2,184	148	6.8	
Dyspnea grade				<0.001
I	9,361	299	3.2	
II	13,656	524	3.8	
III	14,625	793	5.4	
IV	28,156	2,185	7.8	
V	34,890	7,873	22.6	
Unspecified	17,702	5,011	28.3	
Consciousness level				<0.001
Alert	141,110	15,125	10.7	
Dull	19,303	3,841	19.9	
Somnolence	4,763	1,450	30.4	
Coma	4,370	2,344	53.6	
ADL score by Barthel Index				<0.001
100	45,488	2,534	5.6	
75–95	15,729	1,051	6.7	
50–70	22,034	2,144	9.7	
25–45	14,822	2,021	13.6	
0–20	43,791	11,249	25.7	
Pneumonia caused by microbes	53,204	9,432	15.1	<0.001
Aspiration pneumonia	12,650	4,286	25.3	<0.001
Interstitial pneumonitis	8,791	2,862	24.6	<0.001
Pulmonary embolism	1,050	216	17.1	<0.001
Respiratory failure	51,532	11,093	17.7	<0.001
Lung cancer	13,930	4,448	24.2	<0.001
Heart failure	32,748	9,276	16.1	<0.001
Cerebrovascular diseases	7,504	1,394	15.7	<0.001
Liver cirrhosis	1,028	232	18.4	<0.001
Chronic renal failure	4,343	965	18.2	<0.001

*Abbreviations*: *BMI* Body mass index, *ADL* Activities of daily life.

### Adjusted risk factors for in-hospital mortality in patients with COPD

Table 2 shows the results of the logistic regression analyses for all-cause in-hospital mortality. Higher mortality in the COPD patients was associated with older age, male sex, lower BMI, more severe dyspnea, lower level of consciousness, and worse disability of ADL. In addition, higher morality was associated with comorbid diseases at admission, including bacterial or viral pneumonia, respiratory failure, aspiration pneumonia, interstitial pneumonitis, pulmonary embolism, lung cancer, heart failure, cerebrovascular diseases, liver cirrhosis, and chronic renal failure.

**Table 2:** **Adjusted odds ratios of all-cause in-hospital mortality in patients with COPD who required emergency hospitalization**

		Odds ratio	95% confidence interval	p value
Age (yr)	40–49	Reference		
	50–59	1.64	1.07–2.50	0.022
	60–69	1.70	1.14–2.54	0.009
	70–79	2.15	1.45–3.19	<0.001
	80–89	2.53	1.70–3.75	<0.001
	90–99	2.67	1.79–3.98	<0.001
	>100	2.50	1.37–4.58	0.003
Sex	Male	Reference		
	Female	0.63	0.59–0.68	<0.001
BMI	<18.5	1.56	1.48–1.65	<0.001
	18.5–22.9	Reference		
	23.0–24.9	0.73	0.67–0.81	<0.001
	25.0–29.9	0.76	0.68–0.84	<0.001
	>30.0	0.69	0.52–0.92	<0.001
Dyspnea grade	I	Reference		
	II	1.25	1.03–1.53	0.027
	III	1.50	1.23–1.82	<0.001
	IV	2.02	1.69–2.41	<0.001
	V	5.36	4.47–6.42	<0.001
	Unspecified	5.77	4.76–6.99	<0.001
Consciousness level	Alert	Reference		
	Dull	1.30	1.20–1.40	<0.001
	Somnolence	1.64	1.46–1.84	<0.001
	Coma	2.98	2.62–3.39	<0.001
ADL score by Barthel Index	100	Reference		
	75–95	1.07	0.96–1.19	0.251
	50–70	1.38	1.25–1.51	<0.001
	25–45	1.94	1.76–2.15	<0.001
	0–20	3.06	2.78–3.37	<0.001
Pneumonia caused by microbes	Yes	1.18	1.11–1.47	<0.001
	No	Reference		
Aspiration pneumonia	Yes	1.35	1.24–1.47	<0.001
	No	Reference		
Interstitial pneumonitis	Yes	2.86	2.66–3.07	<0.001
	No	Reference		
Pulmonary embolism	Yes	1.53	1.15–2.04	0.004
	No	Reference		
Respiratory failure	Yes	1.32	1.24–1.47	<0.001
	No	Reference		
Lung cancer	Yes	4.14	3.79–4.51	<0.001
	No	Reference		
Heart failure	Yes	1.31	1.23–1.40	<0.001
	No	Reference		
Cerebrovascular diseases	Yes	1.17	1.01–1.34	0.032
	No	Reference		
Liver cirrhosis	Yes	1.99	1.48–2.67	<0.001
	No	Reference		
Chronic renal failure	Yes	1.75	1.52–2.01	<0.001
	No	Reference		

*Abbreviations*: *BMI* Body mass index, *ADL* Activities of daily life.

## Discussion

Our study using a national inpatient database in Japan investigated all-cause in-hospital mortality in 172,707 patients with COPD who required emergency hospital admission. The all-cause in-hospital mortality was 13.7%. Older age, male sex, lower BMI, more severe dyspnea, lower level of consciousness, and worse disability of ADL at admission were significantly associated with higher mortality in these patients. In addition, comorbid diseases at emergency admission, including bacterial or viral pneumonia, aspiration pneumonia, interstitial pneumonitis, pulmonary embolism, respiratory failure, lung cancer, heart failure, cerebral infarction, liver cirrhosis, and chronic renal failure, were associated with higher mortality.

The present study has demonstrated that higher mortality in patients with COPD who required emergency hospitalization was significantly associated with patients’ general conditions at emergency admission. Previous studies reporting the mortality of patients with acute exacerbation of COPD demonstrated that higher mortality in those patients was associated with older age [8, 16, 17], male sex [17, 18], lower BMI [7, 19], severe dyspnea [19, 20], and lower level of consciousness [17, 21, 22]. In the present study, these factors were also predictors of mortality in patients with not only exacerbations of COPD but also all-cause emergency hospital admission.

Our study further demonstrated that disability of ADL scored by the Barthel Index was an important predictor of short-term in-hospital mortality in emergently hospitalized patients with COPD. The Barthel Index has been used as an index for disability of ADL and evaluations, and is known to be one of the risk factors for early unplanned readmission in older patients [23]. The Barthel Index was also reported to be impaired in older patients with COPD [24] and associated with 6-month mortality in older patients with mild COPD [25]. However, there are no reports about the relationship between short-term mortality in emergently hospitalized patients with COPD and the Barthel Index at admission. A previous study by Garcia-Aymerich et al. [26] demonstrated that regular physical activity reduced hospitalization and mortality in patients with COPD, recommended that patients with COPD should be encouraged to maintain or increase their level of regular physical activities, and suggested the importance of physical activities in patients with COPD. Recently, Waschki et al. [27] reported that daily physical activities were a strong predictor of long-term all-cause mortality in patients with COPD, using physical activity levels measured with a multisensory armband. Thus, physical activities in COPD patients should be an important factor affecting both their long-term and short-term mortality.

Comorbid diseases with COPD, including pulmonary and extrapulmonary comorbidities, were also reported to be important for the mortality of patients with COPD [5, 28]. In terms of pulmonary comorbidities, previous studies reported that pneumonic exacerbation of COPD [29], lung cancer [30], and pulmonary embolism [31] were associated with increased mortality in patients with COPD. Our study also demonstrated that these prognostic factors were predictors of mortality in patients with COPD who required emergency admission. In particular, the mortalities for aspiration pneumonia, interstitial pneumonitis, and lung cancer were higher than those of other comorbidities. The coexistence of COPD and pulmonary comorbidities would functionally and pathophysiologically influence one another and might affect the higher mortality. In terms of extrapulmonary comorbidities, heart failure, cerebrovascular diseases, liver diseases, and renal diseases were reported to be independent predictors of mortality in patients with acute exacerbation of COPD [7, 32]. Our study also demonstrated that these comorbidities were predictors of mortality in patients with COPD who required emergency admission, and confirmed the findings of the previous studies.

Several limitations in this study should be acknowledged. The diagnoses of COPD and other comorbidities were based on physician-diagnosed COPD and comorbidities. The accuracy of the diagnosis of COPD was not certified by specialist-diagnosed COPD. However, since COPD is not always diagnosed by specialists in the real-world, the data for physician-diagnosed COPD for studies such as epidemiologic studies and the present study are thought to be meaningful results. In addition, since the DPC database does not include the stages of COPD severity or details of pulmonary function tests, including forced expiratory volume in 1 second and other indices, the degree of airflow limitation was not evaluated in this study. Thus, we cannot discuss the association between mortality of patients with COPD and severity of COPD by airflow limitation. Although airflow limitation is known to be one of the important predictors of mortality, the patients’ general conditions and comorbidities at admission were also independent predictors for emergently hospitalized patients with COPD, independently of the severity of COPD [27]. Moreover, it was recently reported that a former-year exacerbation is a significant predictor of COPD exacerbation [33]. However, evaluation of former-year exacerbations was too difficult in the present study, because patients are not always admitted to the same hospital and it is impossible to identify individuals readmitted to other hospitals in the nationwide DPC database. Therefore, we could not evaluate the former-year exacerbations in this study or discuss the association with mortality.

## Conclusions

In conclusion, our present study investigated all-cause in-hospital mortality in patients with COPD who required emergency hospital admission not only for exacerbations of COPD, but also for any cause. Higher mortality was associated with older age, male sex, lower BMI, more severe dyspnea, lower level of consciousness, and worse disability of ADL on admission. Comorbidities with pneumonia, including aspiration and interstitial pneumonitis, pulmonary embolism, respiratory failure, lung cancer, heart failure, cerebrovascular disease, liver cirrhosis, and chronic renal failure were associated with higher mortality. Therefore, recognition of patients’ general conditions and their comorbidities at admission would be helpful for understanding the all-cause mortality and would provide a strategic direction for choosing better treatment options for COPD patients.

## Abbreviations

COPD: Chronic obstructive pulmonary disease
ADL: Activities of daily life
DPC: Diagnosis procedure combination
ICD-10: International classification of disease and related health problems 10th revision
SD: Standard deviation
BMI: Body mass index.
